# Des Hommes Engagés

**DOI:** 10.1371/journal.pbio.1001722

**Published:** 2013-12-03

**Authors:** Jon Beckwith

**Affiliations:** Department of Microbiology and Immunobiology, Harvard Medical School, Boston, Massachusetts, United States of America

## Abstract

Jon Beckwith reviews Sean Carroll's new book, *Brave Genius*.

[Fig pbio-1001722-g001]In the 1950s, my wife-to-be, Barbara, and I, like many other college students, read and were excited by the works of the French writers Jean-Paul Sartre and Albert Camus. Over the years, we read as many books as we could by and about these two existentialists, their lives and their eventually acrimonious intellectual battles [1]. Early on, we came to consider ourselves existentialists. Just last year we re-read Camus' *The Plague* [2] in French to each other and, on a trip to Aix-en-Provence, went to an exhibit featuring passages of Camus' work that exemplified his striking, consistent, almost painterly use of words to describe colors. Camus, who was engaged with the world around him essentially for his whole life was often described as an *homme engagé* (committed man), a term the French use to describe such intellectuals.

**Figure pbio-1001722-g001:**
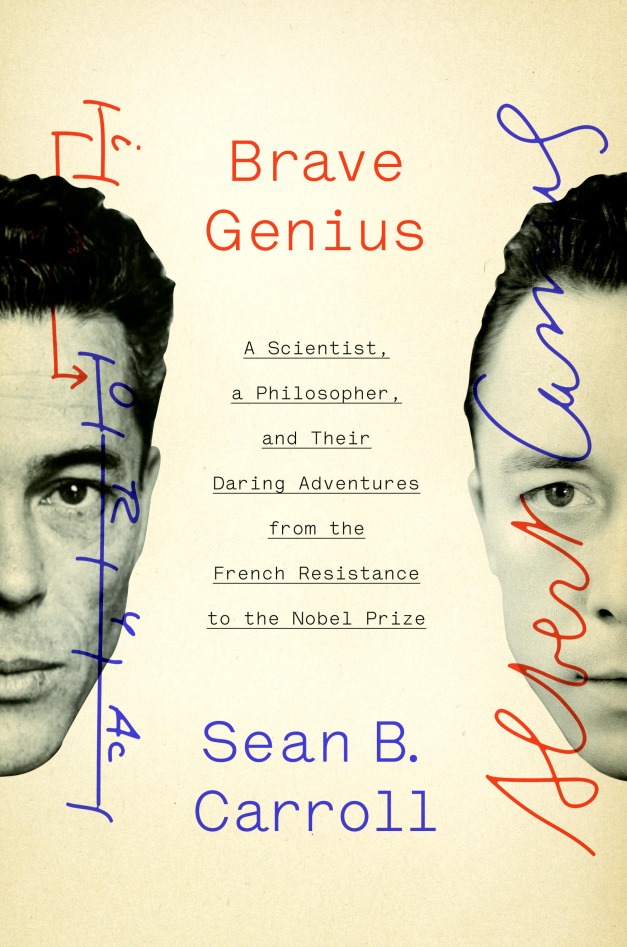
Carroll SB (2013) Brave Genius: A Scientist, a Philosopher, and Their Daring Adventures from the French Resistance to the Nobel Prize. New York: Crown. 576 pp. ISBN 978-0-307-95233-2 (hardcover). US$28.00.

In graduate school, I developed another French-connected passion. The scientific research of *Institut Pasteur* biologists François Jacob, Jacques Monod, Élie Wollman, and their colleagues inspired me to seek a position in their group. I applied and was invited to do postdoctoral work in Jacob's lab for the year 1964–1965. There, in addition to the science with Jacob, I got glimpses, through conversations with members of the lab, of Jacob and Monod's lives outside science. I learned that during World War II Jacob joined de Gaulle's Free French Army and had been badly wounded after the Normandy invasion and that Monod spent his days as a researcher at the *Institut Pasteur* and his nights helping the *Résistance* (the French Resistance movement) fight the German occupiers.

Our year in Paris made us ardent Francophiles; we returned to France again and again. We were curious about the Resistance—about how people make choices that lead them to risk their lives for a cause. We visited at least a dozen museums of the Resistance that recounted the stories and often-tragic fate of its members. We talked with Resistance fighters, including the father of one of Monod's students.

Two months after returning from our recent trip to France, I received a request to review the book *Brave Genius: A Scientist*, *a Philosopher*, *and Their Daring Adventures from the French Resistance to the Nobel Prize* written by evolutionary biologist Sean B. Carroll. The book, catalyzed by Carroll's discovery of a close connection between Monod and Camus, chronicles their lives, as well as Jacob's, from before World War II through much of the post-war period up until Monod's death in 1976. Carroll has, in effect, done the historian's work, ferreting out library, newspaper, *Institut Pasteur* archives, obtaining private letters, and interviewing those still alive who were connected to Jacob and Monod.

It is difficult for me to be objective about *Brave Genius*. It brings together several of my passions, including my fascination with French history and the Resistance. One always learns more with each new book that recounts individual stories of the Resistance, and *Brave Genius* is no different. But *Brave Genius* adds a surprising, provocative finding: the little-known and productive friendship between Camus and Monod. This liaison, since it adds a rare example of a breaching of the “two cultures” [3] barrier, should be of interest to scientists and anyone else who thinks about the interactions between science and society.

It is not surprising that Sean Carroll would write this book about engaged scientists, as he himself publishes occasional columns in the *New York Times* about scientific developments, has entered into battles over the teaching of evolution in schools, and currently heads the Howard Hughes Medical Institute science education program.

The first half of *Brave Genius* chronicles the lives of Monod, Camus, and François Jacob from the beginnings of World War II to the liberation of Paris. Carroll covers Monod's scientific work during this period, along with the writings and developing philosophy of Camus. (Although both Monod and Camus were involved in the Resistance, the two met only after the liberation of France.) During the period of German occupation, Camus wrote some of his most important works, including *The Plague* in which he portrays a city's response to an outbreak of bubonic plague—symbolic of the Nazi occupation and the spread of fascism as well as exploring deeper questions about the human condition. He joined a Resistance group in southern France and then moved to Paris where he became Editor of the important underground Resistance newspaper *Combat*.

Camus' activities during the war are fairly well known. But, little was known publicly about Monod's exploits in the Resistance, which are dramatic enough to have provided the plot-line for one of the many movies and books on the subject. In 1938, Monod, although initially uncertain about continuing in science, began his research career at the Sorbonne, moving to the *Institut Pasteur* five years later. He served in the French army that was confronting the German invasion in 1940 and returned to Paris to join the Resistance when Marshall Pétain surrendered to the Germans. He connected with the network of ethnologists and anthropologists at the *Musée de l'Homme* (Museum of Man) who comprised one of the first Resistance groups in France. Monod faced great danger as he distributed the group's newspaper at night. Members of the network, including close friends, were killed or deported; Monod himself had several close escapes.

With the *Musée de l'Homme* group crushed, Monod joined the communist-led *Franc-Tireurs* (Free Shooters) group where he recruited and trained new members. On one mission, he trekked through the snowy Alps to avoid arrest so that he might reach Geneva, where he was to request money for arms from the United States Office of Strategic Services, the precursor of the present Central Intelligence Agency. After the allied landings at Normandy in 1944, Monod was chosen to prepare battle plans for the *Franc-Tireurs* that would facilitate the success of the allied forces as they approached Paris. He recruited chemist Frédéric Joliot-Curie to provide a recipe for Molotov cocktails.

Remarkably, while Monod had both daytime (research) and nighttime (Resistance) jobs, he also led a Bach chorale group for some time. Despite these “distractions” from science during the war, he and his student Alice Audereau made two important discoveries concerning the ability of bacteria to utilize the sugar lactose as their carbon source.

Carroll tells us not only of Monod's exploits in the Resistance, more extensive than I imagined, but also how he continued his engagement with the post-war world until his death in 1976. In 1948, Monod attracted wide attention with his stinging critique of Lysenkoism, published in *Combat*, which Camus had left the previous year. Trofim Lysenko controlled genetics and agriculture in the Soviet Union from about 1930 to 1964, convincing Stalin of his theories of the heritability of acquired characteristics. Lysenko's power led to the death or exile of a number of geneticists and, some say, to the demise of Soviet agriculture. Monod's critique of the Soviet Union and Lysenko's ideological influence on science policy was a useful example to Camus as he was beginning to find himself in the midst of an increasingly bitter public feud with Sartre over Camus' anti-Soviet positions. In this same year, Monod attended meetings of a group co-founded by Camus that was anti-totalitarian, anti-Stalinist, critical of “American worship of technology,” and closely connected to the periodical *Révolution Prolétairienne*.

After that meeting, Monod and Camus found that they shared many of the same perspectives and they became friends. Carroll argues reasonably that Camus' discussions of scientific matters in his 1951 book *The Rebel* [4] reflected Monod's critique of Lysenkoism and his knowledge of contemporary genetics and evolutionary theory, although there are no citations to support that conclusion. Camus, in a 1957 letter to Monod, said, “we are united in the same adventure.” Monod, whose 1971 book *Chance and Necessity* [5] contains passages that read like existentialist literature, stated subsequently “Camus' existentialism in the widest sense is what I share.” (Camus, who received the Nobel Prize in Literature in 1957, died in a car accident in 1960.) It is clear from their correspondence and the comments of friends of both that Monod and Camus were dear to each other, yet there are no memoirs from either of them that might have given us a fuller picture of this remarkable relationship. (They both died relatively young.) Nevertheless, they were close enough for Camus, in 1949, to ask Monod if he could arrange for medical care for the father of one of his many mistresses, the famous actress Maria Casarès.

After the war, Monod continued to be politically engaged, becoming involved in numerous struggles against injustice. He protested the US government's rejection of visa requests for himself and other Europeans who had been former Communists. His protest letter, published in 1952 in *Science* magazine, caused an international stir. After the Russians crushed the Hungarian uprising in 1956, Monod became the organizer of an ultimately successful effort to smuggle a couple, both scientists, out of Hungary. The extraordinary strategies Monod devised to extricate the two may have benefited from his experience in the Resistance. One of the two scientists, Agnes Ullmann, became his long-time close collaborator. In 1965, Monod, André Lwoff, and Jacob, shortly after receiving word of their Nobel Prizes, publicly called on the French government to support the use of contraception. In 1966, when Martin Luther King visited France to give a fund-raising speech before 5,000 people at Paris' *Palais des Sports*, Monod was asked to introduce him. In 1968, when French students manned the barricades to protest the educational system—protests that led to unrest in much of France—both Monod and Jacob supported the students, supplying them with food and medical supplies. A newspaper photo of Monod shows him helping students wounded by the police.

François Jacob's war experiences also had their dramatic moments as described in Carroll's book, but his own memoir has already described these in great detail [6]. From 1950, at the *Institut Pasteur*, Jacob participated with Monod in some of his more political activities, and also wrote a number of articles for French and US newspapers criticizing eugenics, Nobel Prize laureate sperm banks, and racist theories [7].

In addition to the Camus–Monod friendship and their influence on each other, a growing scientific collaboration between Jacob and Monod flowered in the late 1950s: a collaboration described by Jacob as something like a love affair. In a dizzying few years they and their colleagues, most notably Arthur Pardee, discovered how genes are expressed and how gene expression can be regulated by a repressor (the *lac* repressor binding to the *lac* operon in bacteria). Their findings were among the handful that initiated the biological revolution we are living through today. It was for this work, and the work that led up to it, that Jacob, Lwoff, and Monod were awarded their Nobel Prizes in 1965. Carroll describes the experiments and their implications in language accessible to non-scientists. Similarly, he provides a very clear explanation of Camus' existentialism, which is so central to this story.

Both Monod and Jacob, to my mind, deserve the sobriquet “*l'homme engagé*.” The stories of their commitment to activism are important for young scientists to know of, since the period of war and post-war activism of scientists is over. As the historian of science Jennet Conant said when lamenting the death of anti-nuclear activist and science educator physicist Philip Morison, scientists currently “have become a quiet, docile lot.” [8]. It is encouraging that there is someone like Sean B. Carroll to make us aware of these “*scientifiques* (scientists) *engagés*.”

I have always felt that the best books about science or scientists are those that embed their stories in the history of the times. They can make the history itself seem more tangible and explain much about the scientists in that history. Two recent books of this sort are Kai Bird and Martin Sherwin's *American Prometheus: The Triumph and Tragedy of J. Robert Oppenheimer* and Rebecca Skloot's *The Immortal Life of Henrietta Lacks*. Sean B. Carroll's book *Brave Genius* provides the same kind of enlightenment as these other rich stories.

**About the Author**

Jon Beckwith is Professor Emeritus at Harvard Medical School. His research career has focused mainly on the use of bacterial genetics to study fundamental biological processes. He has published over 300 papers in this field and, among other awards, has received the 2005 Abbott Lifetime Achievement Award of the American Society for Microbiology. He has also been active in issues at the interface of science and society, he teaches a course entitled ‘Social Issues in Biology’ every year and has published over 100 articles on these subjects. He has also published a memoir *Making Genes*, *Making Waves: A Social Activist in Science* (Harvard University Press, 2002). For his work in this latter area, he received the 2009 Edinburgh Medal for “professional achievements that have made a significant contribution to the understanding and well-being of humanity.”
